# Physiological and Psychological Changes Following Liposuction of Large Volumes of Fat in Overweight and Obese Women

**DOI:** 10.15436/2376-0494.15.032

**Published:** 2015-12-09

**Authors:** Allan Geliebter, Emily Krawitz, Tatiana Ungredda, Ella Peresechenski, Sharon Y. Giese

**Affiliations:** 1 Mt. Sinai St. Luke’s Hospital and Dept of Psychiatry, Mt. Sinai School of Medicine, New York; 2 Touro College and University System, New York; 3 New York Eye and Ear Hospital, New York

**Keywords:** Ghrelin, Leptin, Weight loss, Depression, Body image, Fat removal, Cosmetic surgery

## Abstract

**Background:**

Liposuction can remove a substantial amount of body fat. We investigated the effects of liposuction of large volumes of fat on anthropometrics, body composition (BIA), metabolic hormones, and psychological measures in overweight/obese women. To our knowledge, this is the first study to examine both physiological and psychological changes following liposuction of large volumes of fat in humans.

**Method:**

Nine premenopausal healthy overweight/obese women (age = 35.9 ± 7.1 SD, weight = 84.4 kg ± 13.6, BMI = 29.9 kg/m^2^ ± 2.9) underwent liposuction, removing 3.92 kg ± 1.04 SD of fat. Following an overnight fast, height, weight, waist, and hip circumferences were measured at baseline (one week pre-surgery) and post-surgery (wk 1,4,12). Blood samples were drawn for fasting concentrations of glucose, insulin, leptin, and ghrelin. The Body Shape Questionnaire (BSQ), Body Dysmorphic Disorder (BDD) Examination Self-Report (BDDE-SR), and Zung Self-Rating Depression Scale (ZDS) were administered.

**Results:**

Body weight, BMI, waist circumference, and body fat consistently decreased over time (*p* < .05). Glucose did not change significantly, but insulin decreased from wk 1 to wk 12 (*p* < .05). Leptin decreased from baseline to wk 1 (*p* = .01); ghrelin increased but not significantly. Changes in body fat and waist circumference (baseline to wk 1) correlated positively with changes in insulin during that period, and correlated inversely with changes in ghrelin (*p* < .05). BSQ scores decreased significantly over time (*p* = .004), but scores for BDDE-SR (*p* = .10) and ZDS (*p* = .24) did not change significantly.

**Conclusion:**

Liposuction led to significant decreases in body weight and fat, waist circumference, and leptin levels. Changes in body fat and waist circumference correlated with concurrent changes in the adipose-related hormones, insulin and ghrelin (baseline to wk 1), and body shape perception improved. Thus, besides the obvious cosmetic effects, liposuction led to several positive body composition, hormonal, and psychological changes.

## Introduction

Obesity has become an epidemic in the United States and is associated with various comorbidities, such as cardiovascular disease and insulin resistance^[1]^. Although more closely associated with visceral adipose tissue (VAT), insulin resistance is also correlated with subcutaneous adipose tissue (SAT)^[2,3]^. Despite availability of a variety of weight-loss techniques, including exercise, diet, and behavioral therapy, such lifestyle modifications remain a challenge for many, and most individuals regain much of the lost weight over time^[4]^. An alternative to diet-induced weight loss is bariatric surgery, which can lead to marked sustained reductions in body weight and fat. However, it is restricted to clinically severe obese individuals (BMI ≥ 40 without associated comorbidity or BMI ≥ 35 with comorbidity). Another surgical option is to undergo liposuction, which is defined by The American Society of Plastic Surgeons as a procedure that removes excess fat deposits and improves the body contours and proportions^[5]^, which may also yield some potential metabolic benefits.

Results from previous literature have, however, been inconsistent about metabolic changes following liposuction. Giese et al. found that liposuction of large volumes of fat led to a reduction in fasting plasma insulin and systolic blood pressure, along with decreases in body weight and fat mass, but without changes in fasting glucose, leptin, lipid profiles, or resting energy expenditure^[6]^. Two other studies found that liposuction of large volumes of fat decreased insulin resistance^[7,8]^, glucose, and cholesterol^[8]^. A fourth study, however, failed to show that liposuction significantly altered plasma glucose, insulin, insulin resistance, blood pressure, or lipid concentrations despite a 44% decrease of subcutaneous abdominal adipose tissue by volume^[9]^.

The hormone leptin is secreted by adipose tissue, and thus plasma levels are elevated in obese individuals, but these individuals tend to be leptin resistant and do not benefit from leptin’s ability to suppress appetite^[10]^. On the other hand, levels of the orexigenic hormone ghrelin, associated with meal initiation and hunger, are lower in obesity^[11–14]^. Fasting ghrelin usually increases after diet-induced weight loss, but tends to decrease after gastric bypass surgery, which may be a contributing factor to reduced appetite post-surgery^[15]^. In obese male Zucker rats, ghrelin decreased following liposuction^[16]^, similar to decreased ghrelin levels after gastric bypass surgery^[17]^. However, in humans, it has been observed that ghrelin levels generally increase when body fat decreases^[15]^.

Following weight loss, there is a homeostatic drive to restore the lost weight^[18]^, which can be accompanied by behavioral and psychological changes^19,20]^. Weight reduction has sometimes been associated with an increase in depressed mood and preoccupation with body weight, food, and hunger^[21]^. Among those successful at maintaining weight loss, however, psychological scores of depression and psychological distress generally have not increased^[22]^. Such psychological changes have not been investigated in individuals undergoing liposuction of large volumes of fat. In studies following other forms of cosmetic surgery, depression levels did not change post surgery^[23,24]^. About 3–15% of those seeking cosmetic surgery present with mild to severe Body Dysmorphic Disorder (BDD)^[25–28]^, a negative obsession with a part of the body. Cosmetic surgery did not improve BDD based on clinical interviews^[29]^ or BDD questionnaire scores^[30]^, and did not help prevent the development of BDD in those with mild or sub-threshold BDD^[31]^. These studies, however, did not examine the effects of liposuction on BDD^[30]^. BDD has been shown to be overrepresented in those seeking liposuction^[32]^, suggesting a need to assess BDD following liposuction of large volumes of fat.

Body shape dissatisfaction is generally greater in obese than lean women but can also serve as a motivator for weight loss^[33,34]^. Interventions to reduce body weight, including bariatric surgery, improved body image in the overweight and obese^[33–39]^. Furthermore, there is some evidence that body image improves following obesity treatment, independent of the degree of weight loss^[40,41]^.

Although there are studies on the psychological effects of weight loss and cosmetic surgery, there are none examining the psychological effects of the removal of large volumes of fat in overweight and obese individuals. In the present study, we examined changes in both physiological and psychological aspects following liposuction of large volumes of fat in overweight and obese women who had approximately 4 kg of subcutaneous fat tissue removed. To our knowledge, this is also the first study to measure ghrelin levels in humans following liposuction. We hypothesized that glucose, insulin, and leptin levels would decrease and ghrelin levels would increase following liposuction. We also expected that scores of depression, body dysmorphic disorder, and body shape dissatisfaction would improve.

## Materials and Methods

### Subjects

Nine obese and overweight women (BMI = 29.9 ± 2.9 SD) underwent liposuction removing a large volume of fat (Table-1). Four participants had more than four liters (L) of fat removed, considered large volume liposuction (LVL), four participants had just under 4L of fat removed and one participant had 2.3 L removed, with a range of fat aspirate of 2.3 – 5.9L. All women were premenopausal, and were not pregnant or using birth control. They had no history of cardiac, renal, gastrointestinal or endocrine disease, including diabetes. Patients were recruited by the surgeon (SG) over a seven-month period from the regular pool of patients visiting the office and signed an approved IRB consent form. Their racial and ethnic distribution, based on self-report, was 22% white, 22% Hispanic, 44% black, and 11% other. Patients were not requested to change their eating or exercise habits prior to or after surgery.

### Liposuction

All patients underwent subcutaneous adipose tissue (SAT) liposuction of the abdomen. A super wet technique was used, with small volumes of fluid injected into the fatty deposits prior to surgery to facilitate fat removal. In order to prevent excessive bleeding, dilute lidocaine and epinephrine were injected into the SAT. Several patients additionally had small amounts of SAT removed from their backs, flanks, arms, inner and outer thighs for additional cosmetic benefits. An internal ultrasound- assisted liposuction system with a 4-mm hollow bullet- tip probe was used. This was followed by suction, using a Mercedes cannula with a diameter of 4 mm and a length of 15 mm, containing three lateral suction holes. The volume of fat aspirate was measured by collecting the aspirate in a container during suction, and allowing the fat to rise to the top and weighed 30 min later which yielded 4358 cc ± 1155 SD. Following surgery, patients were dressed in special compression garments. No post-surgical adverse effects were reported and inflammation was absent by 12 weeks.

### Materials

Patients were tested at baseline (1 week prior to surgery), and at wk 1, wk 4, and wk 12 following surgery. Fasting blood samples were collected at each visit to measure plasma insulin, glucose, leptin, and ghrelin. Blood samples were centrifuged, and plasma separated and stored in microtubes at −80 °C until analyzed. Glucose was assayed with a Beckman glucose analyzer (glucose oxidase method), insulin with a radioimmunoassay (RIA) kit from Linco (intra-assay CV = 5.5, inter-assay CV = 4.9), leptin with an RIA kit from Linco (intra-assay CV = 4.8, inter-assay CV = 5.9), and total ghrelin (acylated and desacylated) was assayed with an (RIA) from Phoenix Pharmaceuticals (intra-assay CV = 5.2, inter-assay = 6.2). Insulin resistance was estimated with the homeostasis model assessment (HOMA) from fasting glucose and insulin concentrations: fasting insulin (µU/ml)×fasting glucose (mg/dL)/405. Patients completed the Body Shape Questionnaire (BSQ)^[42]^, a measure of weight and shape concern and dissatisfaction. They also completed the Body Dysmorphic Diagnostic Examination Self-Report (BDDE-SR), a questionnaire that assesses the degree of Body Dysmorphic Disorder (BDD)^[43]^ based on obsession with a specific, most distressing body part. Finally, they completed the Zung Self-Rating Depression Scale (ZDS)^[45]^, selected due to its advantages of reverse scoring and assessment of suicidal ideation.

### Sample size

Sample sizes were estimated from the most related studies for power = 0.80, using effect size Cohen’s d (G*Power 3.1.) with 2-tailed α = 0.05. From a related study on biological changes (6), following large volume liposuction, insulin decreased from 14.9 (mIU/ml) ± 6.5 SD pre-surgery to 7.2 ± 3.2, 4 months post-surgery. Based on this, d = 1.37, and the minimum sample size n= 7 would be needed. From a related study^[46]^ on psychological changes, the Body Areas Satisfaction Scale (BASS) scores improved from 3.15 ± 0.64 SD to 3.50 ± 0.61, d= 1.19, and minimum sample size n = 9. Hence, nine subjects were considered adequate.

### Statistics

Repeated measures ANOVA was performed over the four time periods. The ANOVA was checked for sphericity, and the degrees of freedom were adjusted when appropriate. When the overall F was significant (*p* < .05), post-hoc paired tests were performed. Two tailed *p* values < .05 were considered significant, and p values ranging from .05 to .08 were considered to be a trend. Means and standard deviations (SD) are used in the text and tables; means and standard errors (SEM) are used in the figures. Missing data were interpolated as follows: When the baseline data was missing, it was not interpolated, and thus the data for that subject was not used. When the missing data were between two known values, the average was used for interpolation. If the data was missing from week 12, or from both week 4 and week 12, the last observation carried forward (LOCF) method was used. There were missing data for waist circumference and body fat measurements, and for BDD, Zung, and BSQ questionnaires, which represented 18% of the total number of possible data for those variables. None of the hormone or glucose values were missing. SPSS (IBM SPSS, Chicago, IL) was used to analyze the data.

## Results

### Anthropometric measurements

**Figure 1:**
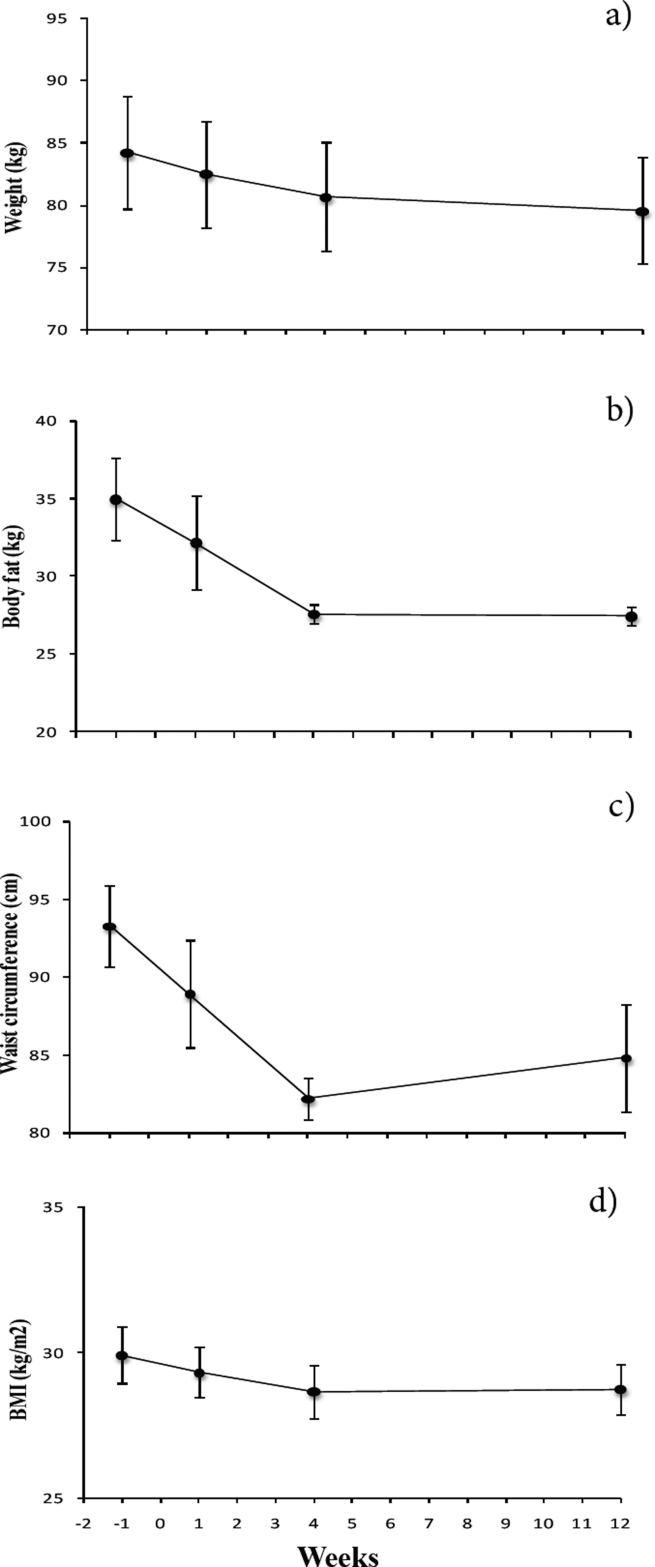
a) Body weight (n = 9) decreased significantly between all time points (F_3,24_ = 20.2, *p* < .001; post hoc, all *p*’s < .03) following liposuction. b) Body fat decreased significantly (F_3,24_ = 13.0, *p* < .001) from preop baseline to wk 1, 4, and 12 (post hoc, all *p* ‘s < .01). c) Waist circumference decreased significantly between all time points (F_3,24_ = 44.5, *p* < .001; post hoc, all *p’s* < .03). d) BMI decreased significantly (F_3,24_ = 4.3, *p* = .02) from baseline to wk 1 and to wk 4 as well as wk 1 to wk 4 (post hoc, *p* < .03). A trend was shown from baseline to wk 12 (post hoc, *p* = .06).

Body weight decreased significantly between all time points (F_3,24_ = 20.2, *p* < .001; post hoc, all *p*’s < .03). From baseline to wk 12, patients had a mean weight reduction of 4.7 ± 2.8 kg (Figure 1a). BMI decreased significantly (F_3,24_ = 4.3, *p* = .02) from baseline to wk 1, baseline to wk 4, and wk 1 to wk 4 (post hoc, *p* = .03, .001, and .003, respectively), and from baseline to wk 12 (*p* = .06, trend). Body fat decreased significantly (F_3,24_ = 13.0, *p* < .001) from baseline to wk 1, baseline to wk 4, and baseline to wk 12 (post hoc, *p* = .01, .01, and .003, respectively). Waist circumference decreased significantly over all time points (F_3,24_ = 44.5, *p* < .001; post hoc all *p*’s < .03). All the data above are shown in Figure 1.

### Glucose and Hormones

**Figure 2:**
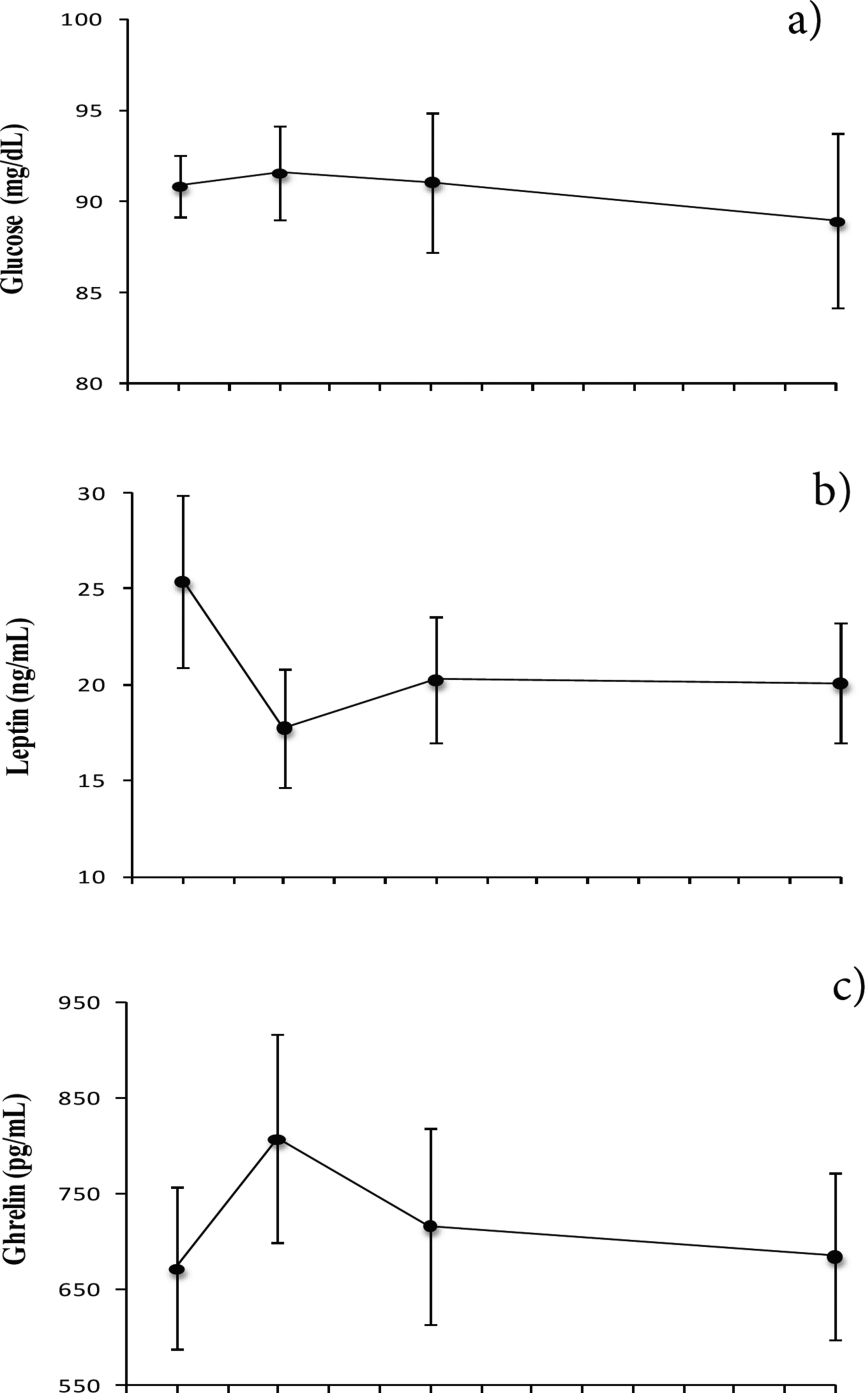

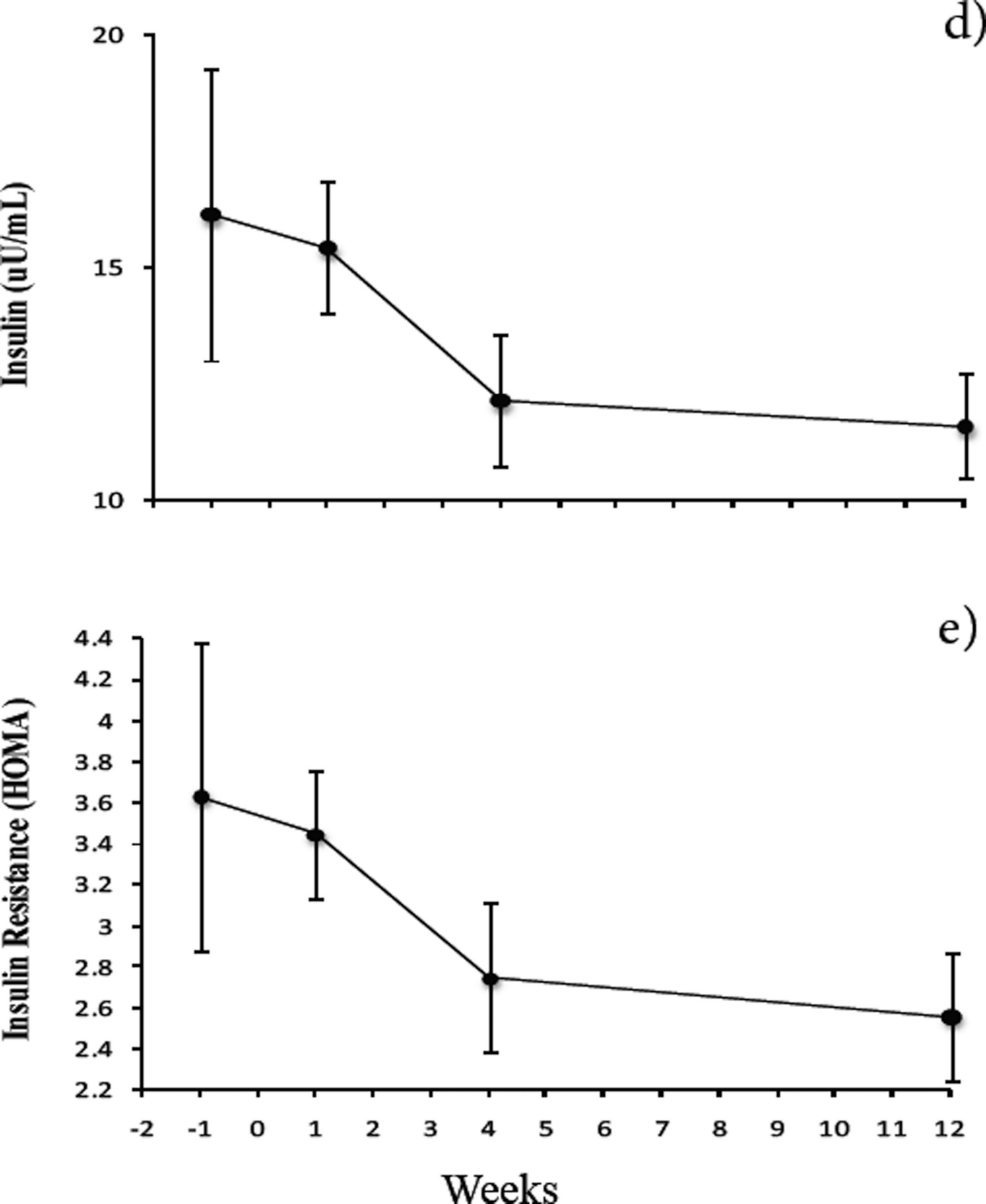
a) Glucose did not change significantly (F_3,24_ = 0.2, *p* = 0.89). b) Leptin decreased significantly (F_3,2_4 = 4.1, p = 0.02) from baseline to wk 1(post hoc, *p* = .01), and a trend from baseline to wk 4 and to wk 12 (post hoc, *p* = .08 and *p* = .08, respectively). c) Ghrelin did not change significantly (F_3,24_ = 1.7, *p* = .19), but it showed a trend to decrease from wk 1 to 12 (post hoc, *p* = .06). d) Insulin decreased significantly from wk 1 to 12 (F_3,24_ = 1.4, *p* = 0.27; post hoc, *p* = .049). e) Insulin resistance (HOMA) did not change significantly (F_3,24_ = 1.16, *p* = 0.35).

Fasting glucose levels (F_3,24_ = 0.2, *p* = 0.89) and insulin resistance (HOMA) (F_3,24_ = 1.16, *p* = 0.35) did not change significantly over time (Figure 2a,e). Insulin levels decreased significantly from wk 1 to wk 12 (F_3,24_ = 1.4, *p* = 0.27; post hoc, *p* = .049) (Figure 2d). Changes between baseline and wk 1 in body fat correlated positively with changes in insulin (*r* = 0.7, *p* = .03) and inversely with changes in ghrelin (r = −0.8, *p* = .02). Furthermore, changes in waist circumference between baseline and wk 1 correlated with changes in insulin (*r* = 0.9; *p* = .01), changes in leptin (r = 0.9; *p* = .004), and changes in glucose (*r* = 0.8; *p* = .05). There was an overall significant decrease in leptin levels (F_3,24_ = 4.1, *p* = 0.02) (Figure 2b). Post hoc tests revealed significant differences from baseline to wk 1 (*p* = .01), and trends from baseline to wk 4 (*p* = .08) and baseline to wk 12 (*p* =. 08). Ghrelin levels did not change significantly (F_3,24_ = 1.7, *p* = .19), but showed a trend to decrease from wk 1 to wk 12 (*p* =. 06) (Figure 2c). Ghrelin and leptin correlated inversely with each other at baseline (*r* = −0.73, *p* = .03) and at wk 1(*r* = − 0.79, *p* = .01).

The hormone data were then reanalyzed using weight change as a covariate in the repeated measures ANOVA. The previous changes in insulin and leptin levels were no longer significant. When the amount of fat removed was entered as a covariate, the hormonal changes in leptin and insulin were also no longer significant. Furthermore, the covariates (weight change and fat removal) were not significant predictors on their own.

### Psychological Measures

**Figure 3:**
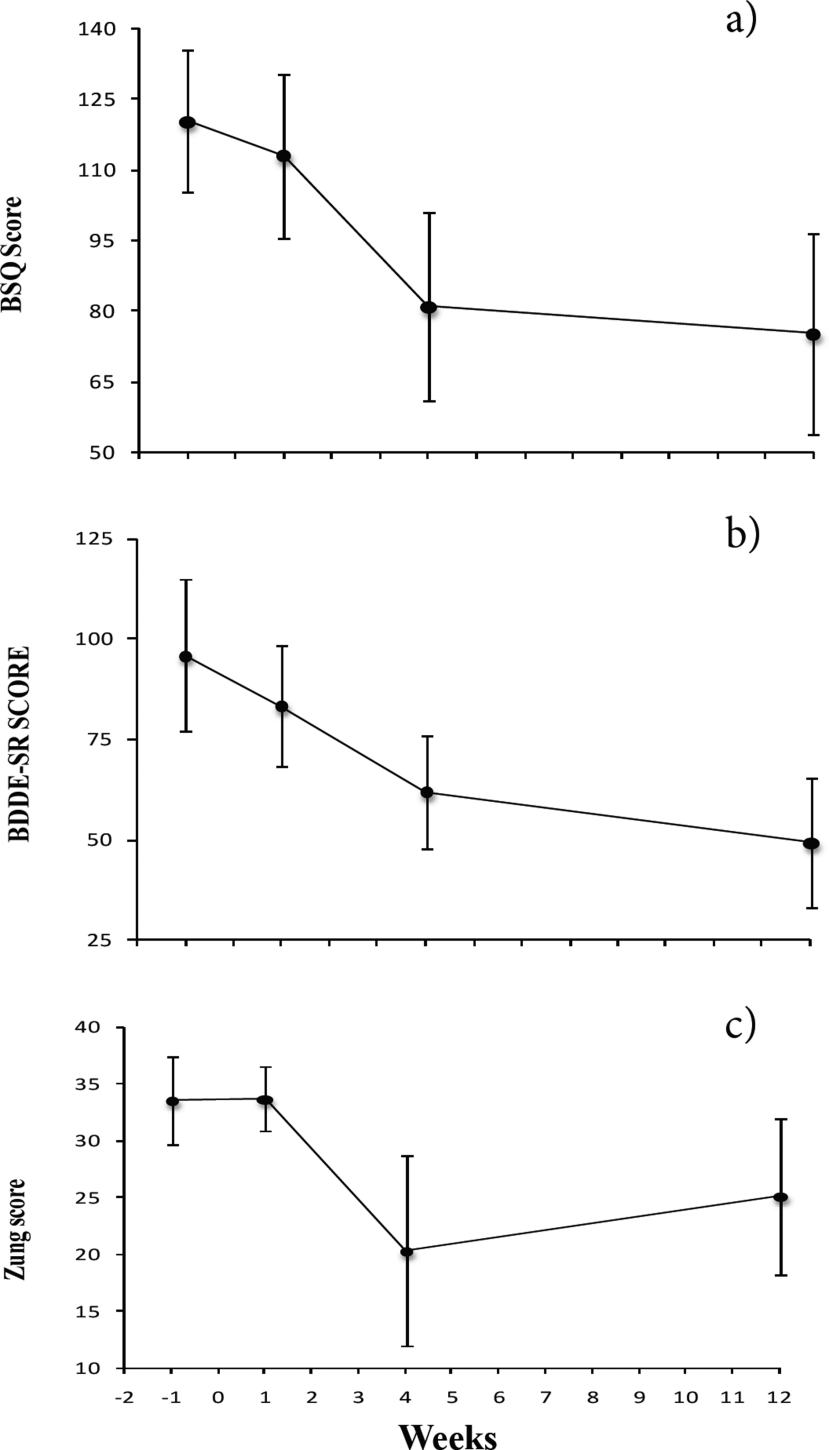
a) Body Shape Questionnaire scores declined significantly (F_3,18_ = 6.4, *p* = 0.004) from baseline to wk 4 (post hoc, *p* = .002), and to wk 12 (post hoc, *p* = .03). b) Body Dysmorphic Disorder Examination Self-Report (BDDE-SR) scores did not change significantly (F_3,18_ = 2.4, *p* = 0.1). c) Zung depression scores did not change significantly (F_3,18_ = 1.5, *p* = 0.24).

Body Shape Questionnaire (BSQ) scores improved significantly (F_3,18_ = 6.4, *p* = 0.004) from baseline to wk 4 and baseline to wk 12 (post hoc, *p* = .002 and *p* = .03, respectively) (Figure 3a). Body Dysmorphic Disorder scores (BDDE-SR) and Zung depression scores (ZDS) did not change significantly (F_3,18_ = 2.4, *p* = 0.1 and F_3,18_ = 1.5, *p* = 0.24, respectively) (Figure 3b,c; Table 2). When repeated measures ANOVA for BSQ scores were performed with weight change or fat removal as covariates, the results were no longer significant, and both covariates were not significant predictors on their own.

## Discussion

The results showed that liposuction of large volumes of fat led to a significant decrease in plasma leptin levels from baseline to wk 1, and a significant decrease in plasma insulin from wk 1 to wk 12, although plasma glucose, insulin resistance (HOMA), and plasma ghrelin did not change significantly. When weight change and amount of fat removed were entered as covariates, the significance was lost. This suggests that both weight change and amount of fat removed had an influence on leptin and insulin levels although the covariates did not influence the hormone levels significantly on their own.

Results from other studies indicate some similarities and differences with the present results. A study on overweight (BMI 27–30, age 39.4 ± 6.8 SD) women who underwent liposuction of large volumes of fat revealed that they had significant decreases four months postoperatively in fasting plasma insulin, insulin resistance, and body weight and fat mass, but no significant changes in glucose or leptin^[6]^. Giugliano et al. found that six months post liposuction of large volumes of fat, insulin resistance (HOMA) was significantly reduced in obese and normal- weight women, which correlated with the amount of fat removed^[7]^. Robles-Cervantes et al. reported that three weeks post liposuction of large volumes of fat, 15 non-obese women had significant reductions in glucose and cholesterol, with increases in insulin secretion, whereas insulin resistance (HOMA-IR) remained unaltered^[8]^. Klein et al., separated women into two groups, those with type 2 diabetes (n = 7; age, 52 ± 3) and those with normal glucose tolerance (n = 8; age = 42 ± 3) and did not find that liposuction altered plasma glucose, insulin, or insulin resistance significantly 10–12 weeks after surgery^[9]^. However, a number of plasma collections were made at 10 weeks, which may be too short a time period to observe changes in insulin.

D’Andrea et al. studied metabolic changes following liposuction of large volumes of fat in 123 healthy women with grade 1 obesity^[47]^. They found that 90 days post-operatively, there were significant decreases in fasting plasma insulin, 2-h plasma glucose, insulin resistance, and plasma leptin, and that each factor was correlated with a decrease in fat mass and waist-to-hip ratio. These authors also found an improvement in oxidative and non-oxidative glucose metabolism, as well as lipid oxidation, which they suggested as the reasons for improved insulin sensitivity. It is possible that we did not find an improvement in insulin resistance in our study due to the relatively large variance in insulin levels in our subjects at preoperative baseline.

We found that the largest change in the appetite-related hormones leptin and ghrelin occurred at wk 1, and that the decrease in fat at wk 1 correlated both with leptin (positively) and ghrelin (inversely). This is consistent with previous findings that body fat correlates positively with leptin and negatively with ghrelin^[15]^. Our study was the first to look at ghrelin levels following liposuction of large volumes of fat in humans, and we found no significant changes.

### Psychological Effects

Liposuction of large volumes of fat led to lower scores in BSQ at wk 4 and wk 12 post-surgery, reflecting improvement in body image. Commonly, body image improves with weight loss^[33–39]^. Roughan et al. showed significant improvements in body image when women lost an average of 2.8 kg over 10 weeks and another 1 kg at 2 year follow-up^[33]^. In a study by Foster et al., body image improved overall following a 48-week weight-loss program^[35]^. However, from week 24 to 48, participants experienced a small weight gain, which was associated with a small, but significant worsening in body image. Other recent studies also showed that weight loss was associated with body image improvement^[36–38]^. In our study, after controlling for weight change and amount of fat removed, we found that both had a partial influence on body dissatisfaction but were not significant predictors on their own.

We did not observe a change in depression scores, which may in part be due to the absence of high baseline scores, which would reflect clinical depression. The lack of change in depression scores is consistent with a postsurgical study for breast reduction^[24]^, in which scores of depression and anxiety did not change^[24]^. Similarly, following a variety of cosmetic surgeries, negative thoughts, quality of life, and depression did not change at nine months^[23]^.

In our study, scores reflecting the degree of BDDE-SR also did not change significantly. In a review by Gipson et al. on various types of cosmetic surgery (rhinoplasty, breast reduction or enlargement, blepharoplasty, and hair transplants), BDD did not improve post-surgery^[29]^. Furthermore, in a prospective rhinoplasty study, those who presented with mild BDD pre-surgery still had BDD five years post-surgery^[48]^. Similarly, in one cosmetic surgery study on seven patients with BDD, six presented with BDD five years postoperatively^[31]^. In contrast in another study, women undergoing a variety of cosmetic procedures showed decreased BDDE-SR scores six months postoperatively^[30]^. It is possible that this decrease was due to the women being instructed to answer the BDDE-SR, specifically in reference to the feature to be altered rather than the feature that most distressed them, which is the standard protocol^[30]^. Results in the present study showed that liposuction, similar to other cosmetic surgeries, did not improve BDDE-SR.

Limitations of this study include a relatively small number of participants, although the power calculation suggested the number would be adequate, and the lack of a control group.

In conclusion, this was the first study to examine both physiological and psychological effects of liposuction of large volumes of fat. We found that liposuction in overweight and obese women significantly reduced body weight, BMI, waist circumference, body fat, and plasma leptin and insulin levels. However, it did not significantly change glucose or ghrelin concentrations. Moreover, changes in body fat correlated with concurrent changes in levels of the adipose-related hormones, insulin, and ghrelin from baseline to wk 1. Furthermore, changes in waist circumference correlated with concurrent changes in levels of the adipose-related hormones, insulin and leptin, as well as glucose from baseline to wk 1. Liposuction of large volumes of fat also led to improvement in body image. Thus, liposuction led to several positive body composition, hormonal, and psychological changes.

## Figures and Tables

**Table 1:** Preoperative Anthropometric Measures in Nine Overweight Women

Measure	Range	Mean ± SD
**Age (y)**	23 – 46	35.9 ± 7.1
**Preoperative Weight (kg)**	66.9 – 110.5	84.3 ± 29.0
**Height (m)**	1.6 – 1.8	1.7 ± 0.1
**Body Mass Index (kg/m^2^)**	26.7 – 36.0	29.9 ± 2.9
**Waist Circumference (cm)**	86.0 – 112.0	95.6 ± 9.2
**Hip Circumference (cm)**	96.0 – 132.0	109.3 ± 11.5

**Table 2:** Psychological Measures at Baseline and Postop wk 1, 4, 12 (mean ± SD)

	Baseline	wk 1	wk 4	wk 12
BSQ	120.4 ± 40.3	110.5 ± 43.1	96.75 ± 34.3	92.1 ± 43.3
BDDE-SR	82.1 ± 50.2	71.4 ± 39.3	56.9 ± 27.6	51.1 ± 36.5
ZDS	33.6 ± 10.3	35.3 ± 8.2	32.6 ± 11.1	33.1 ± 12.3

BSQ, Body Shape Questionnaire BDDE-SR, Body Dysmorphic Disorder Examination Self-Report ZDS, Zung Self-Rating Depression Scale
